# Roots of the Malformations of Cortical Development in the Cell Biology of Neural Progenitor Cells

**DOI:** 10.3389/fnins.2021.817218

**Published:** 2022-01-05

**Authors:** Chiara Ossola, Nereo Kalebic

**Affiliations:** Human Technopole, Milan, Italy

**Keywords:** neural progenitor and stem cells, neurogenesis, cortical malformation, neocortex, neuronal migration

## Abstract

The cerebral cortex is a structure that underlies various brain functions, including cognition and language. Mammalian cerebral cortex starts developing during the embryonic period with the neural progenitor cells generating neurons. Newborn neurons migrate along progenitors’ radial processes from the site of their origin in the germinal zones to the cortical plate, where they mature and integrate in the forming circuitry. Cell biological features of neural progenitors, such as the location and timing of their mitoses, together with their characteristic morphologies, can directly or indirectly regulate the abundance and the identity of their neuronal progeny. Alterations in the complex and delicate process of cerebral cortex development can lead to malformations of cortical development (MCDs). They include various structural abnormalities that affect the size, thickness and/or folding pattern of the developing cortex. Their clinical manifestations can entail a neurodevelopmental disorder, such as epilepsy, developmental delay, intellectual disability, or autism spectrum disorder. The recent advancements of molecular and neuroimaging techniques, along with the development of appropriate *in vitro* and *in vivo* model systems, have enabled the assessment of the genetic and environmental causes of MCDs. Here we broadly review the cell biological characteristics of neural progenitor cells and focus on those features whose perturbations have been linked to MCDs.

## Human Cortical Development

One of the most intriguing features that characterizes the human species is the exceptional size of the cerebral cortex, in particular the neocortex. Humans show a significant expansion in both radial and tangential direction of the cerebral cortex, which is involved in the increased cognitive abilities that we consider unique to humans. This expansion is a consequence of the increased neuronal production during the embryonic and fetal development, due to a prolonged neurogenic period and to an increased proliferative capacity of different neural progenitor cell types (Rakic, 2009; Geschwind and Rakic, 2013; Wilsch-Bräuninger et al., 2016; Sousa et al., 2017; Molnar et al., 2019; Kalebic and Huttner, 2020; Pattabiraman et al., 2020; Del Valle Anton and Borrell, 2021). In addition, the survival of newborn neurons also plays a role, as the inhibition of apoptosis was shown to increase brain size (Kuida et al., 1996, 1998; Rakic and Zecevic, 2000; Roth and D’Sa, 2001).

During mammalian evolution, the neocortex, which constitutes much of the cerebral cortex, shows the most significant expansion (Kriegstein et al., 2006; Lui et al., 2011; Borrell and Reillo, 2012). The developmental organization of the mammalian neocortex has been described by Rakic (1988, 2000, 2009) in radial unit and protomap hypotheses: it consists of columns positioned tangentially to the cortical surface, generated by distinct proliferative units in the germinal zones (GZs), that form specialized regions with specific cytoarchitecture and function, called areas. Newborn excitatory neurons hence originate from progenitor cells located in the GZs and they migrate to their final location in the cortical plate (CP) along the processes of progenitor cells. In the radial dimension, the adult neocortex is organized in six layers (I-VI). The neocortex is built in an “inside-out” manner, with the deep layers (V–VI) generated first and the upper layers (II–IV) following subsequently (Angevine and Sidman, 1961; Rakic, 1988; Bayer and Altman, 1991; Molyneaux et al., 2007). The layer I, however, is formed by the earliest-born neurons that form a preplate which later splits into the marginal zone populated by Cajal-Retzius cells (Marin-Padilla, 1978; Zecevic and Rakic, 2001; Bielle et al., 2005) and the subplate (Kostovic, 2020). In contrast to excitatory neurons, cortical inhibitory interneurons are generated in the medial and caudal ganglionic eminences and undergo a tangential migration to reach the developing neocortex (Hu et al., 2017).

**FIGURE 1:**
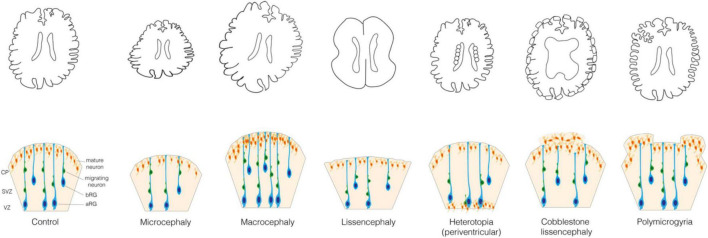
Malformations of the cortical development. **(Upper)** Schematic representations of the control brain and brains affected by the following MCDs: microcephaly, macrocephaly, lissencephaly, periventricular nodular heterotopia, cobblestone lissencephaly and polymicrogyria. **(Lower)** Schematic representation of the mechanisms underlying these MCDs. In Control: VZ, ventricular zone; SVZ, subventricular zone; CP, cortical plate; aRG, apical radial glia; bRG, basal radial glia; migrating and mature neurons.

Developing mammalian brain exhibits apicobasal polarity with the apical side facing the lumen of the ventricles and the basal side facing the skull. The developing neocortex contains two principal GZs: the ventricular zone (VZ), situated along the ventricle, and the subventricular zone (SVZ), which is located more basally between the VZ and the intermediate zone (IZ). The latter is the layer through which newborn neurons migrate along the progenitors’ scaffold toward the cortical plate (CP) (Figure 1). In contrast to lissencephalic mammals, whose brain is smooth, such as mouse, the gyrencephalic mammals, whose brain is folded, such as ferrets and primates, exhibit a massively enlarged SVZ that contains two cyto-architectonically specific sublayers, the inner and outer SVZ (iSVZ and oSVZ), separated by an axon-rich fiber layer (Smart et al., 2002; Reillo and Borrell, 2012; Dehay et al., 2015; Saito et al., 2018). Whereas the primate iSVZ is comparable to the mouse SVZ, with densely packed cells, the oSVZ cells have a radial arrangement similar to the VZ (Smart et al., 2002; Dehay et al., 2015).

The differences in neocortex size and complexity across mammals are widely considered to derive from the differences in the proliferative capacity and neurogenic potential of neural progenitor cells (Lui et al., 2011; Fernandez et al., 2016; de Juan Romero and Borrell, 2017; Molnar et al., 2019; Kalebic and Huttner, 2020). Neuroepithelial cells (NECs) are the first progenitor cells specifically devoted to neocortical development and the source of all other neocortical progenitors (Taverna et al., 2014). Before the onset of neurogenesis, NEC population is enlarged by symmetric proliferative divisions, which is prominent in primates, due to an extended period of proliferation (Rakic, 1995). At the beginning of neurogenesis NECs undergo asymmetric divisions to generate apical Radial Glia (aRG), a new pool of progenitor cells that replaces the NECs and populates the VZ (Figure 1). aRG maintain the epithelial apicobasal polarity, with an apical process that lines the ventricle and tightly seals the tissue with an adherens junction belt, and a basal process that contacts the basal lamina and provides a scaffold fundamental for the radial migration of newborn neurons to the CP (Rakic, 2003; Taverna et al., 2014). aRG can undergo self-amplifying symmetric proliferative divisions that increase their population and asymmetric divisions to produce more differentiated progenitor cell type called Basal Progenitors (BPs), or rarely a neuron. BPs delaminate from the apical belt of adherens junctions at the surface of the VZ and migrate basally to form the SVZ (Haubensak et al., 2004; Miyata et al., 2004; Noctor et al., 2004).

Basal Progenitors include two subtypes: basal intermediate progenitors (bIPs) and basal or outer Radial Glia (bRG or oRG) (Figure 1) (Taverna et al., 2014; Kalebic and Huttner, 2020). In species with a small and smooth brain, such as mouse, BPs usually divide only once to generate 2 neurons, whereas in species with an expanded and folded neocortex, such as ferret and primates, BPs have a greater proliferative capacity and they can undergo several proliferative divisions producing other BPs before finally generating neurons. This is particularly relevant for the bRG, whose abundance (up to 50% of all BPs) and proliferative capacity are significantly increased in humans, macaques and ferrets, compared to lissencephalic mammals (in mouse, bRG comprise up to 10% of BPs), suggesting a role in neocortical expansion (Fietz et al., 2010; Hansen et al., 2010; Reillo et al., 2011; Betizeau et al., 2013; Lewitus et al., 2014; Kalebic et al., 2019). Whereas the basal processes of both bRG and aRG are crucial for neuronal migration, bRG processes are considered particularly important for the tangential dispersion of newborn neurons, a key feature of folded brains (Borrell and Götz, 2014; Fernandez et al., 2016; Nowakowski et al., 2016; Kalebic and Huttner, 2020).

The complexity that characterizes human cortical development makes it particularly vulnerable to the effects of genetic mutations and environmental factors. The resulting alterations of the cortical development can lead to cortical malformations, defects that are considered to be a probable cause for neurological conditions such as epilepsy, autism spectrum disorder or intellectual disability (Pan et al., 2019).

## Malformations of Cortical Development

Malformations of cortical development are a heterogeneous group of disorders characterized by macroscopic alterations in the brain structure caused by genetic mutations or environmental factors that affect neocortical development (Sun and Hevner, 2014; Bizzotto and Francis, 2015; Desikan and Barkovich, 2016; Romero et al., 2018; Juric-Sekhar and Hevner, 2019; Subramanian et al., 2019; Klingler et al., 2021). Such alterations of brain structure can include abnormal brain size, layering, folding and presence of heterotopic gray matter. As the causative genetic mutations are highly diverse, the classification of MCDs is typically based on the neurological outcomes, whereas the diagnosis is mostly based on MRI data (Barkovich et al., 2012; Guarnieri et al., 2018; Severino et al., 2020). Three major groups of MCDs can be identified based on the affected developmental phase: (i) cell proliferation and survival, (ii) neuronal migration, (iii) post-migrational differentiation and circuits connectivity (Barkovich et al., 2012; Romero et al., 2018; Subramanian et al., 2019; Severino et al., 2020; Francis and Cappello, 2021).

When cell proliferation or survival are affected, neurogenesis can be increased or reduced, which leads to an increase or reduction of the brain volume, that is megalencephaly or micrencephaly, respectively (Homem et al., 2015; Jayaraman et al., 2018). This in turn usually leads to a consequent modification of the entire head dimension, that is macrocephaly and microcephaly, respectively (Figure 1). Microcephaly is characterized by a reduction in the total number of neurons generated developmentally and is typically caused by a decreased neocortical progenitor pool size or an abnormal apoptosis. The former is accompanied by a premature differentiation of neurons and can most often be caused by various abnormalities of the mitotic phase and neural progenitor polarity (Jamuar and Walsh, 2015; Jayaraman et al., 2018). Instead, macrocephaly is typically characterized by a localized increase in the number or size of neurons and glial cells, which is usually due to mutations in key signaling pathways that regulate cell proliferation and growth (Mirzaa and Poduri, 2014; Hevner, 2015).

A disruption of the migration of newborn neurons from GZs to CP can lead to an aberrant localization of neocortical neurons and a failure in formation of cortical layers (Buchsbaum and Cappello, 2019; Castello and Gleeson, 2021). Such disruption can be a consequence of the primary defect in the migrating neuron or in the progenitor cell (aRG or bRG) whose processes are providing a scaffold for the migrating neurons. In this review we focus on the cell biology of neural progenitors. MCDs such as heterotopia and lissencephaly can arise when the neuronal migration is incomplete, whereas polymicrogyria and cobblestone lissencephaly are usually observed in relation to an excessive migration (Figure 1) (Francis and Cappello, 2021). Heterotopia is characterized by clusters of normal neurons in abnormal locations (Barkovich et al., 2012; Guerrini and Dobyns, 2014; Guarnieri et al., 2018) whose migration was arrested in different moments. In case of periventricular nodular heterotopia (Figure 1), the nodules or thick bands of gray matter are stuck at or near the ventricular surface, whereas in subcortical heterotopia newborn neurons remain in the white matter (Barkovich et al., 2012; Guerrini and Dobyns, 2014). Lissencephaly (“smooth brain”, also known as classical or type I lissencephaly) is characterized by an abnormal gyral pattern accompanied by thickening and altered layering of the neocortex (Di Donato et al., 2017; Buchsbaum and Cappello, 2019). It includes a spectrum of conditions, ranging from broadening of few gyri, as in the case of pachygyria, to a complete absence thereof, as in the case of agyria. The latter, however, is extremely rare as it is observed almost only in Miller–Dieker syndrome (Blazejewski et al., 2018). Polymicrogyria refers to an overfolding of the neocortex with presence of abnormally small gyri that can be focal, multifocal, or affecting the entire neocortex, often characterized by dyslamination with displaced and disoriented neurons and discontinuities in the pial basement membrane (Figure 1) (Squier and Jansen, 2014; Stutterd and Leventer, 2014; Jansen et al., 2016; Diamandis et al., 2017; Sarnat and Flores-Sarnat, 2021). Cobblestone lissencephaly (also known as type II lissencephaly) shows a cobblestone-like brain surface due to an over-migration of neurons through ‘gaps’ in the pial basement membrane (Figure 1) (Devisme et al., 2012; Buchsbaum and Cappello, 2019). The MCDs characterized by incomplete or excessive migration are most often caused by mutations in genes related to cytoskeleton and cell adhesion (Moon and Wynshaw-Boris, 2013; Jamuar and Walsh, 2015; Romero et al., 2018).

The final step of neocortical development is the post-migrational differentiation with integration into neuronal circuits, which includes complex events like growth and maturation of axons and dendrites, synaptogenesis and synaptic pruning. MCDs arising at this stage usually lead to certain forms of focal cortical dysplasia (FCD) and dysgyria. FCD is characterized by disorganized cortical lamination, variability in cortical thickness and abnormal gyral pattern (Najm et al., 2018; Subramanian et al., 2019). Dysgyria refers to a dysmorphic cortex with abnormalities in gyral size and sulcal depth (Mutch et al., 2016; Romaniello et al., 2018). The main identified causes of MCDs affecting post-migrational differentiation and integration into neuronal circuits are mutations in signaling molecules and cytoskeletal proteins (Jamuar and Walsh, 2015; Manikkam et al., 2018; Romaniello et al., 2018).

The MCD-causing mutations often arise *de novo* during gametogenesis or postzygotic development (Wilfert et al., 2017; Juric-Sekhar and Hevner, 2019). The timing of mutation is often associated with the severity of phenotype (Sarnat, 1987; Sarnat and Flores-Sarnat, 2014). In this context, somatic mutations have recently been found to exert and important contribution to MCDs with focal insult, such as in focal heterotopia, focal cortical dysplasia and hemimegalencephaly (Jamuar et al., 2014; Jansen et al., 2015; Gonzalez-Moron et al., 2017; Montier et al., 2019). Somatic mutations can be of type 1 or type 2, which cause a new heterozygous mutation or lead to a loss of heterozygosity, respectively (Qin et al., 2010; Jansen et al., 2015; Juric-Sekhar and Hevner, 2019).

The landscape of pathological conditions of MCDs is highly heterogeneous, as are the genetic variants and molecular pathways involved in the disease onset. However, the classifications of MCDs are mainly based on the neurological outcome and neuroimaging data. Furthermore, specific genetic mutations or events can impact the cortical development by affecting different cell types and/or different developmental phases, thus causing various MCDs (Sapir et al., 2019; Klingler et al., 2021). In this review we focus on the neural progenitor cells and their role in generation of neurons and supporting neuronal migration. To bridge across scales from genetic mutations to neurological outcomes, we focus on the cell biological level, as a key interaction point between genes and phenotypes.

## Cell Biological Basis of Malformations of Cortical Development in Neural Progenitors

Below we discuss the cell biological features of neural progenitors involved in the onset of MCDs. We particularly focus on the progenitor proliferation and polarity as key aspects disrupted in MCDs. Tight regulation of mitosis is the key cell biological feature influencing the proliferation of neural progenitors. Hence, protein products of many MCD-causative genes are associated to the mitotic spindle. Various microtubule-associated proteins that operate at centrosome, kinetochore or that are involved in microtubule dynamics and severing have been implicated with MCDs. Similarly, actin-binding proteins that are important for the progression of mitosis and its fine regulation are also associated with MCDs. To correctly progress through the cell cycle, neural progenitors need to be able to receive extrinsic signals and to transduce this information *via* intracellular signaling pathways. Maintaining correct cell polarity is a fundamental cell biological feature that enables both the exposure to extrinsic signals and the neuronal migration. Various molecules operating in both the apical and the basal endfeet of aRG and bRG, adherens junctions, cell trafficking, cilium as well as components of the extracellular matrix and its receptors have been implicated in MCDs. Further, different receptors and molecules operating in pro-proliferative signaling pathways are often mutated in MCDs. Finally, several metabolic enzymes, transcription factors and epigenetic modifiers that promote proliferation of neural progenitors are found to be mutated in patients with MCDs. Considering that many molecules, whose mutations are associated with MCDs, operate in multiple cell biological domains, we typically discuss individual molecules within the cell biological context that is the most relevant for the disease aetiology.

### Mitotic Spindle

**FIGURE 2:**
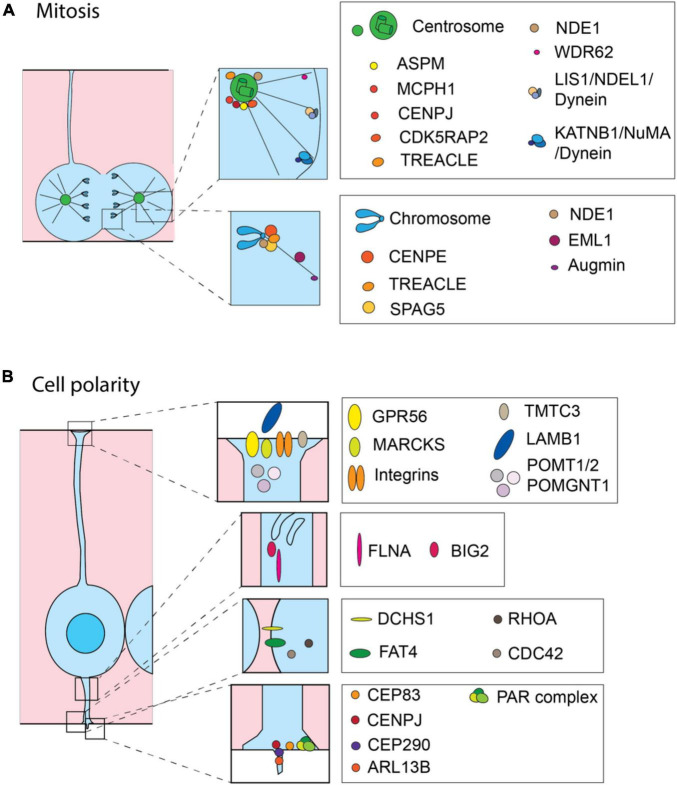
Cell biological processes and molecules involved in the onset of MCDs. Schematic representation of proteins involved in MCDs. Most of the proteins are related to the precise regulation of mitosis **(A)** and maintaining correct cell polarity **(B)**. **(A)** During mitosis the MCD-involved proteins are mainly operating at the centrosome and astral microtubules (upper inset) or at the kinetochore (lower inset). **(B)** In interphase, MCD-involved proteins are operating at the basal endfoot (first inset from the top), intracellular trafficking (second inset from the top), adherens junctions (third inset from the top) and apical endfoot including cilium (the lowest inset) to maintain cell polarity.

The mitotic spindle is the key element involved in establishing the orientation of the cleavage plane, that is symmetric versus asymmetric cell division, and therefore is involved in determining the fate of the daughter cells (Lancaster and Knoblich, 2012; Taverna et al., 2014; Matsuzaki and Shitamukai, 2015; Kalebic and Namba, 2021). Hence, it is not surprising that the proteins implicated in the function of the mitotic spindle are often found mutated in microcephaly (Bond and Woods, 2006; Sun and Hevner, 2014) (Figure 2A). A notable example is ASPM (abnormal spindle-like microcephaly-associated protein), whose mutations are the most common cause of primary microcephaly (Bond et al., 2002; Thornton and Woods, 2009). Depletion of Aspm from mouse cortex results in a reduction of vertical symmetric proliferative division of apical progenitors (NECs and aRG) and subsequent depletion of the progenitor pool (Fish et al., 2006; Pulvers et al., 2010). However, the phenotypes observed in mutant mice were poorly recapitulating the extent of microcephaly in human patients (Pulvers et al., 2010) likely because mouse neural progenitors are less proliferative than the human progenitors. Interestingly, a knockout (KO) of *Aspm* in the developing ferret neocortex led to a premature detachment of aRG and generation of bRG, which in turn led to a severe microcephaly, similarly to what was observed in human patients (Johnson et al., 2018).

The second most common cause of primary microcephaly are mutations in WDR62 (WD repeat domain 62, also known as MCPH2), a scaffold protein associated with the spindle pole (Bilguvar et al., 2010; Nicholas et al., 2010). WDR62 mutants fail to localize to the spindle pole and cells transiently arrest in mitosis (Nicholas et al., 2010; Farag et al., 2013; Chen et al., 2014; Sgourdou et al., 2017). Depletion of Wdr62 in mice lead to microcephaly due to reduced proliferation of neural progenitors, spindle instability and abnormalities in centrosome inheritance (Chen et al., 2014; Sgourdou et al., 2017). Mechanistically, WDR62 has been shown to interact with JNK1/2 (c-Jun N-terminal kinase) and AURKA (Aurora kinase A) as well as AURKB (Aurora kinase B), which is likely regulating its localization to the spindle pole and cell cycle progression in neocortical progenitors (Chen et al., 2014; Lim N. R. et al., 2015; Sgourdou et al., 2017). Mutations in MCPH1 (microcephalin 1) are another notable cause of primary microcephaly (Jackson et al., 2002). Disruption of *Mcph1* in mice leads to a mitotic spindle misalignment and a premature shift from symmetric to asymmetric cell divisions (Gruber et al., 2011). Importantly, MCPH1 is further involved in chromatin condensation and DNA damage repair with effects that span beyond the neocortex (Zhou et al., 2013; Pulvers et al., 2015; Houlard et al., 2021).

Various genes encoding for centrosomal proteins were found mutated in primary microcephaly (Figure 2A) (Bond et al., 2005; Gilmore and Walsh, 2013; Bizzotto and Francis, 2015). CDK5RAP2 (Cyclin Dependent Kinase 5 Regulatory Subunit Associated Protein 2; MCPH3) and CENPJ (Centromere protein J; MCPH6) are two notable examples (Bond et al., 2005). CDK5RAP2 mutant mice show thinning of the cerebral cortex with a reduction in late-born neurons, as a consequence of an early depletion of the progenitor pool due to the abnormal orientation of the mitotic spindle which leads to increased horizontal and reduced vertical cleavage planes (Lizarraga et al., 2010). CENPJ deletion in mice embryo also leads to a microcephaly, which in this case is a consequence of a progressive loss of centrioles in aRG and the subsequent detachment of progenitor cells from the VZ. Those detached progenitors continue proliferating, but exhibit a mitotic delay, which leads to an induction of apoptosis and a subsequent reduction in the number of progenitors (Insolera et al., 2014).

Similarly, *TCOF1*/TREACLE (Treacher Collins syndrome protein) is a centrosome- and kinetochore-associated protein that is required for mitotic progression and its mutation have been identified in patients with Treacher Collins syndrome who can exhibit microcephaly (Sakai et al., 2012). *Tcof1* mutant mice show abnormal spindle orientation, increased asymmetric cell division and defects in cell proliferation resulting in a smaller progenitor pool, fewer neurons and decreased brain size (Sakai et al., 2012). CENPE (Centromere protein E) is a core kinetochore component required for spindle microtubule capture and attachment at the kinetochore (Abrieu et al., 2000; Yao et al., 2000). Mutations in CENPE alter spindle dynamics and chromosome segregation leading to delayed mitotic progression and finally resulting in a microcephaly with a simplified gyral pattern (Mirzaa et al., 2014b).

During mitosis microtubules are generated by the centrosomal nucleation, chromatin-mediated nucleation and by nucleation from the surface of other microtubules (Meunier and Vernos, 2016). The latter mechanism is mediated by augmin that binds to the microtubule lattice, upon its generation by the centrosome- and chromatin-dependant pathways, and promotes the growth of new microtubules as branches (Kamasaki et al., 2013). Conditional knockout of the augmin subunit Haus6 in aRG leads to spindle assembly defects, p53-mediated apoptosis and an increase in DNA damage, which in turn leads to a reduction of neurogenesis, disruption of tissue integrity and finally an abortion of the brain development (Viais et al., 2021).

Mutations in genes that encode for proteins involved in microtubule dynamics can also lead to abnormal spindle orientation and subsequent MCDs (Figure 2A). EML1 (echinoderm microtubule associated protein-like 1) is a microtubule-associated protein whose mutation leads to complex cortical malformations characterized by megalencephaly with a ribbon-like heterotopia and callosal agenesis (Kielar et al., 2014; Oegema et al., 2019). The spontaneously arisen mutant mice (HeCo mice) and KO mouse models recapitulate the heterotopia phenotype (Kielar et al., 2014; Collins et al., 2019). HeCo mice are characterized by a perturbation of microtubule dynamics and an increase in oblique cleavage orientations of aRG, that lead to ectopic proliferation of progenitor cells in IZ and CP (Kielar et al., 2014; Bizzotto et al., 2017). NDE1 (nudE neurodevelopment protein 1), whose mutations in human patients can lead to a severe microcephaly with lissencephaly, is involved in centrosome duplication and mitotic spindle assembly, attachment of microtubules to kinetochores and proper neuronal migration (Feng and Walsh, 2004; Vergnolle and Taylor, 2007; Alkuraya et al., 2011). Its ablation in the mouse neocortex induces microcephaly, due to abnormal orientation of cleavage plane, aberrant chromosome localization, mitotic delay and premature cell cycle exit (Feng and Walsh, 2004). Similarly, mutations observed in patients truncate the C-terminal domain, which is involved in the localization to the centrosome (Alkuraya et al., 2011). Consequently, they dysregulate cytoskeletal dynamics in mitosis, leading to spindle-structure defects that include tripolar spindles, misaligned mitotic chromosomes, nuclear fragmentation and abnormal microtubule organizations (Alkuraya et al., 2011). Deletion or mutations of LIS1 (Lissencephaly 1 protein; encoded by *PAFAH1B1*, Platelet activating factor acetylhydrolase 1b regulatory subunit 1) are the major cause of classical lissencephaly in humans (Reiner et al., 1993; Romero et al., 2018). Different rodent models have shown that LIS1 is important for both progenitor proliferation and neuronal migration (Cahana et al., 2001; Gambello et al., 2003; Tsai et al., 2005). LIS1 deletion leads to proliferation defects of apical progenitors due to misorientation of the mitotic spindle, reduced and weakened astral microtubules and increased apoptosis (Yingling et al., 2008). Mechanistically, Lis1 operates with Nde1 to target the cytoplasmic dynein complex, whereas the complex Lis1/Ndel1 (Nde1 like)/dynein is important for microtubule stability and cortical capture (Feng and Walsh, 2004; Yingling et al., 2008; McKenney et al., 2010; Moon et al., 2020). Lis1 and dynein are connected to the nuclear envelope through their interaction with the SUN- and KASH-domain proteins, SUN1/2 and Syne/Nesprin-1/2, which have a critical role in nuclear movement during neurogenesis and neuronal migration, as their ablation in mice leads to defects in cortical lamination and reduced brain size (Zhang et al., 2009).

Another protein involved in microtubule dynamics at mitosis, KATNB1 (B1 subunit of the microtubule severing enzyme katanin), has been associated with severe microcephaly with lissencephaly (Mishra-Gorur et al., 2015). KATNB1 plays an important role at the spindle pole together with cytoplasmic dynein and NuMA (nuclear mitotic apparatus protein), which tethers spindles at the poles and is fundamental for microtubule aster formation (Mishra-Gorur et al., 2015; Jin et al., 2017). Studies of various proteins involved in spindle positioning in both *Drosophila* neuroblasts and mammalian neocortical progenitors suggest an important role of spindle orientation for determination of cell fate, notably in generation of BPs (Lancaster and Knoblich, 2012; Matsuzaki and Shitamukai, 2015). For example, overexpression of Insc (Inscuteable) or a KO of LGN lead to an increase in BPs, that in case of LGN KO exhibit characteristics of bRG (Konno et al., 2008; Postiglione et al., 2011). Considering the importance of BPs, and in particular bRG, for the human cortical development, it is important to examine the mechanisms of bRG generation in order to better understand the MCD aetiology.

### Actin Cytoskeleton and Cell Division

Several actin-binding proteins implicated in MCDs operate during the cell division of neural progenitors. Mutations in FLNA (Filamin A), which crosslinks actin filaments and links them to membrane proteins, can be a cause of periventricular heterotopia (Fox et al., 1998; Sheen et al., 2005). Knockout of FLNA in mice leads to prolongation of the cell cycle length by delaying the onset and progression through mitosis due to impaired regulation of cyclin B1 degradation which results in a decline in the progenitor numbers and a reduction of the brain size (Lian et al., 2012). Other actin-binding proteins were shown to be implicated in the neural progenitor division. For example, depletion of n-cofilin, which is involved in depolymerization of actin filaments, leads to an increased cell cycle exit of neural progenitors and a depletion of the progenitor pool (Bellenchi et al., 2007). PFN1 (Profilin-1) regulates actin polymerization and is associated with Miller–Dieker syndrome which is characterized by a lissencephaly (Kwiatkowski et al., 1990). In mutant mice model, absence of PFN1 leads to a change in cleavage plane orientation with an increase in horizontal divisions (Kullmann et al., 2020). This in turn led to an increase in bRG abundance and neuronal production during mid-neurogenesis and a formation of rudimentary neocortical folds. It hence remains important to examine the role of PFN1 and other actin-binding proteins for the proliferation of BPs in the developing human neocortex. Interestingly, a small GTPase Rac1 (Ras-related C3 botulinum toxin substrate 1), which regulates many cellular processes including cytoskeletal organization, is required for survival of neural progenitors and for the normal proliferation and differentiation of BPs, specifically (Leone et al., 2010). Deletion of Rac1 in telencephalon results in microcephaly with reduced size of both cerebral cortex and striatum (Chen et al., 2009), whereas its ablation in the progenitors situated in medial ganglionic eminence impairs their transition from G1 to S phase and leads to an impairment of GABAergic interneurons migration into the cortex (Vidaki et al., 2012).

Actin rearranges at the cell cortex and enhances cell membrane rigidity, which is important for proper anchorage of astral microtubules (Heng and Koh, 2010). DIAPH3 (Diaphanous three) is a member of the formin protein family that nucleate and elongate actin filaments and has a role in spindle assembly and cytokinesis. Two clinical studies point to *DIAPH3* as an autism susceptibility gene (Vorstman et al., 2011; Xie et al., 2016). DIAPH3 deficiency in mice induces centrosome abnormalities, disrupts the spindle and astral microtubules and leads to a loss of cortical progenitors, microcephaly, and autistic-like behavior (Lau et al., 2021). Depletion of DIAPH3 leads to downregulation of SPAG5, a kinetochore- and centrosome-associated protein that controls sister chromatid cohesion and recruitment of CDK5RAP2 to centrosome (Kodani et al., 2015). Knockdown of SPAG5 induces similar mitotic errors as the depletion of DIAPH3 and its overexpression rescues DIAPH3 knockdown phenotype (Kodani et al., 2015; Lau et al., 2021), emphasizing the importance of cooperation of actin and microtubule associated proteins in mitotic progression.

### Cytokinesis

Cytokinesis is the final step in the mitosis. Cleavage furrow of aRG starts on the basal side and ingresses toward the apical membrane, a process which is mediated by anillin and actin-based cortex (Kosodo et al., 2008; Kosodo and Huttner, 2009). The midbody is formed at the end of the furrow as a structure that contains compacted microtubules. The midbody often relocates to the daughter cells containing the apical processes and is subsequently released into the ventricle (Dubreuil et al., 2007; Ettinger et al., 2011). Among the proteins involved in abscission are members of Kinesin-6 family. Interestingly, one of them, Kif20b, was found to be mutated in *magoo* mouse mutant which exhibits a fully penetrant microcephaly (Janisch et al., 2013). Kif20b mutants show changes in the midbody number, shape and position resulting in the disruption of the abscission step, which in turn causes apoptosis of neural progenitors and leads to a small cerebral cortex (Janisch et al., 2013).

### Interphase Centrosome and Cilia

During interphase centrosomes are positioned in the apical endfeet of aRG, where they contribute to cell polarity, ciliogenesis and cell attachment (Taverna et al., 2014). Centrosome consists of two centrioles and upon a cell division, the older centriole is typically inherited by the proliferative daughter cell that remains an aRG, whereas the daughter centriole is inherited by the differentiating daughter cells (neuron or BP) (Wang et al., 2009). Previously mentioned microcephaly proteins ASPM and WDR62 during interphase localize to mother centrioles where they physically interact (Jayaraman et al., 2016). Loss of either of them or both leads to a defective centriole duplication and impaired cilia, resulting in premature delamination of progenitors and microcephaly (Jayaraman et al., 2016). It was shown recently that the mother centriole in aRG contains distal appendages that anchor it to the apical membrane and that CEP83 (centrosomal protein 83), a protein implicated in intellectual disabilities, is required for their maintenance (Figure 2B) (Shao et al., 2020). The elimination of CEP83 led to expansion of the progenitor pool which resulted in macrocephaly with abnormal folding. The rigidity of the apical membrane was affected, and the mechanically sensitive YAP (yes-associated protein) was activated, hence promoting progenitor proliferation (Shao et al., 2020). YAP was shown to promote proliferation of BPs in humans and ferrets, and it would hence be interesting to explore a possible role of this mechanism in human MCDs pertinent to BPs (Kostic et al., 2019). Importantly, AKNA, another protein that associates to mother centrioles, was shown to play a role not only in generation of BPs, but also in retaining cells in the SVZ, which again suggests to examine the role of centrosome-associated proteins in the context of BP cell biology (Camargo Ortega et al., 2019).

The mother centriole forms the basal body, a structure that makes the base of the primary cilium, an organelle involved in signaling, mechanotransduction and cell fate (Taverna et al., 2014). Interestingly, the ciliary membrane, which is endocytosed at the onset of mitosis along with the mother centriole, also tends to be inherited by the proliferative daughter cell (Paridaen et al., 2013). Perturbations of ciliary structure or function leads to ciliopathies such as Joubert and Bardet–Biedl syndromes, which can result in cortical disorganization and intellectual disabilities (Taverna et al., 2014; Bizzotto and Francis, 2015). Mutation in centrosomal proteins CENPJ and CEP290 (centrosomal protein 290) have been associated with ciliary phenotypes (Figure 2B). CEP290, which can be mutated in Joubert syndrome, is required for the function of Rab8, a protein involved in ciliogenesis (Valente et al., 2006; Kim et al., 2008). CENPJ has an important role in regulating cilia disassembly as its loss causes alterations in length of cilia, leading to delayed cell cycle, reduced cell proliferation, and increased cell apoptosis, all together resulting in microcephaly (Ding et al., 2019). ARL13B, mutated in Joubert syndrome, is a small GTPase specifically enriched in the cilium and required for ciliary structure and function. Knockout mice show inverted cell polarity with the cell body located on the pial surface and the basal endfoot positioned on the ventricular side (Higginbotham et al., 2013).

### Cell Polarity

Cell polarity is a fundamental feature of neural progenitor cells. Whereas aRG exhibit a classical apicobasal polarity, with the apical pole lining the ventricular surface and the basal side contacting the pia, BPs show a greater heterogeneity in their cell polarities (Taverna et al., 2014; Kalebic et al., 2017). bIPs typically exhibit multipolar morphology in interphase, whereas bRG show polarized morphology, often containing the basal process contacting the pia, but not the apical polarity domain which can hence be termed pseudo-apicobasal polarity (Kalebic and Namba, 2021).

Apical progenitors contain a specialized apical polarity complex, which is fundamental for cell polarity, and it contains Par3 (Partitioning defective protein 3), Par6 (Partitioning defective protein 6), and aPKC (atypical protein kinase C) (Costa et al., 2008; Bultje et al., 2009; Hansen et al., 2017; Kalebic and Namba, 2021). Par-complex proteins are important for regulating aRG proliferation and differentiation in the developing cerebral cortex, as the inheritance of the apical domain is associated with maintenance of stemness. Par complex contributes to regulating Notch signaling by enriching Notch on the plasma membrane of the daughter cells that inherited the apical domain and thereby enables that cell to maintain its stemness (Bultje et al., 2009; Kandachar and Roegiers, 2012). Overexpression of both Par3 and Par6 promoted generation of proliferative progenitors in the developing mouse neocortex (Costa et al., 2008). Loss of Par3 in developing mouse cortex causes severe MCD including increased volume and massive ribbon-like heterotopia (Liu et al., 2018). This was caused by temporally different progenitor behaviors in response to HIPPO and NOTCH signaling. During early phase of neurogenesis, the progenitor proliferation was increased at the expenses of production of deep-layer neurons, whereas at later stages the differentiation was increased leading to an increased production of upper layer neurons (Liu et al., 2018). The megalencephaly and heterotopia phenotypes were rescued by a simultaneous removal of the HIPPO pathway effectors YAP and TAZ (transcriptional coactivator with PDZ-binding motif), underlining again an important role of HIPPO pathway in cortical development (Liu et al., 2018).

### Adherens Junctions

Adherens junctions play a key role in maintaining aRG polarity and VZ cohesion. Their disruption or instability can affect aRG morphology and lead to detrimental consequences on the cortical lamination and neuronal migration (Figure 2B) (Veeraval et al., 2020). The conditional knockout mouse of N-cadherin, a key junctional protein, displays a complete loss of cortical organization with mitotic and postmitotic cells scattered throughout the cortex and aRG that could not grow their processes (Kadowaki et al., 2007). Deletion of α-E-catenin in mouse cerebral cortex causes an uncoupling of adherens junctions with the intracellular actin fibers, which results in a loss of tissue polarity and formation of large subcortical band heterotopia, as migrating neurons fail to reach the cortical plate and accumulate in ectopic positions (Schmid et al., 2014). Similarly, inactivation of CDH2 (cadherin 2) or afadin, a junctional adaptor protein, results in a disruption of adherens junctions and an increase in progenitor proliferation, which again leads to a phenotype resembling subcortical band heterotopia (Gil-Sanz et al., 2014; Rakotomamonjy et al., 2017). Maintenance of cadherin-based adherens junctions requires Numb and Numbl (Numb-like) which localize to the apical end-foot. Their inactivation disrupts adherens junctions, causes premature progenitor depletion, abnormal progenitor dispersion and disorganized cortical lamination (Li et al., 2003; Petersen et al., 2004; Rasin et al., 2007).

Mutations in the receptor-ligand cadherin pair DCHS1 (Dachsous cadherin-related 1) and FAT4 (FAT Atypical Cadherin 4) have been associated with the Van Maldergem syndrome, which is characterized by a periventricular neuronal heterotopia (Figure 2B) (Cappello et al., 2013). Knockdown of those genes in the developing mouse neocortex results in increased progenitor cell numbers and reduced neuronal differentiation, which in turn leads to a heterotopic accumulation of cells below the neuronal layers (Cappello et al., 2013). Cerebral organoids derived from induced pluripotent stem cells (iPSCs) of patients with mutations in DCHS1 and FAT4 recapitulate the heterotopic phenotype, however, without a change in progenitor proliferation, highlighting the inter-species differences (Klaus et al., 2019). The phenotype of mutant organoids is due to a combination of changes in the morphology of neural progenitor cells and altered navigational system of some neurons, underscoring the importance of the neural progenitor cell biology for the neuronal migration also in the human model system (Klaus et al., 2019).

Rho GTPases are fundamental regulators of cytoskeleton organization and cell polarity during neocortical development. Conditional deletion of RhoA (Ras homolog family member A) in neural progenitors leads to the disruption of apical anchoring and mis-orientation of aRG processes which results in heterotopia underneath a thinner cortex, reminiscent of cobblestone lissencephaly (Cappello et al., 2012). Conditional deletion of Cdc42 (Cell Division Cycle 42) in mice leads to a gradual loss of adherens junctions, impairing the apically directed interkinetic nuclear migration, which results in progenitors undergoing mitoses at basal positions and acquiring the neurogenic fate of BPs (Cappello et al., 2006). Further, mice deficient in both DIAPH1 and 3, actin nucleators and Rho effectors, develop a periventricular dysplastic mass due to disruption of apical adherens junctions in aRG and impaired cell polarity (Thumkeo et al., 2011). Interestingly, a mutation in RhoA activator, PLEKHG6 (Pleckstrin homology and RhoGEF domain containing G6), was found in human heterotopia and it leads to the loss of a primate-specific isoform of this protein (O’Neill et al., 2018). Modulation of PLEKHG6 isoform in human cerebral organoids once again highlights the relevance of BPs and in particular bRG for the aetiology of human MCDs. Similarly, downregulation of another plecstrin homology domain protein, PLEKHA7 (Pleckstrin homology domain containing A7), which is adherens junction belt-specific, results in delamination of neural progenitors from the ventricular surface and conversion of aRG to bRG (Tavano et al., 2018). Furthermore, LGALS3BP (Galectin 3 binding protein), a secreted protein that interacts with components of the extracellular matrix, has been found mutated in patients with altered local gyrification and cortical thickness (Kyrousi et al., 2021). Analysis in electroporated mouse embryos and human organoids suggests that LGALS3BP, which is involved in the apical anchoring, is mediating BP delamination that in turn is critical for the proper neuronal migration and cortical folding (Kyrousi et al., 2021).

### Intracellular Trafficking

Cell polarity is highly dependent on traffic through the secretory pathway and, interestingly, the pathological endoplasmic reticulum (ER) and the Golgi apparatus stress have been associated with MCDs, in particular microcephaly (Figure 2B) (Passemard et al., 2019). Some patients affected by microcephaly and periventricular heterotopia present mutations in the *ARFGEF2* (ADP-ribosylation factor guanine nucleotide-exchange factor-2), encoding for BIG2 [brefeldin A (BFA)-inhibited GEF2 protein]. BIG2 promotes the activation of ARF (ADP-ribosylation factor) proteins by guanine-nucleotide exchange, regulating Golgi vesicular budding and uncoating. *In vitro* experiments show that inhibition of BIG2 decreases cell proliferation of mouse neural progenitors, possibly due to the aberrant intracellular localization of E-cadherin and β-catenin (Sheen et al., 2004). The other principal gene implicated in periventricular heterotopia, FLNA, is also implicated in intracellular trafficking, as it has been shown that FLNA facilitates trafficking of β1 integrin to the cell membrane (Kim et al., 2010). Further, deletion of BIG2 promotes phosphorylation of FLNA which in turn affects FLNA-actin binding affinity and changes the localization of FLNA, suggesting a cooperative action between actin and vesicle trafficking in the assembly of membrane proteins (Zhang et al., 2012, 2013). Considering the recently revealed novel features of the Golgi apparatus in apical versus basal progenitors and the differential contribution of Golgi to the apical versus basal process of the neural progenitors (Taverna et al., 2016; Taverna and Huttner, 2019), it is important in the future to better assess the importance of intracellular trafficking for the onset of MCDs.

### Basal Attachment

Migration of newborn neurons from the site of their generation in GZ to their final position in the CP occurs along the basal processes of aRG and bRG that make a radial scaffold (Rakic, 2009; Geschwind and Rakic, 2013; Fernandez et al., 2016). In lissencephalic species, such as mouse, this scaffold is simpler, it consists mainly of the basal processes of aRG, as bRG are very rare in mouse, and it enables a radial migration of newborn neurons. In gyrencephalic species, such as human, ferret or macaque, this scaffold is more complex and apart of the radial migration, it enables also the tangential dispersion of neurons, which is required for correct folding (Fernandez et al., 2016; Kalebic and Huttner, 2020; Del Valle Anton and Borrell, 2021; Kalebic and Namba, 2021). Such complexity might be facilitated by the existence of bRG morphotypes with two basal processes, which were described in humans and ferrets, but not in mice (Kalebic et al., 2019). Further, at mid-neurogenic period human aRG become truncated, loose their basal processes and the migration of the late-born neurons is mediated only by the bRG-generated scaffold (Nowakowski et al., 2016).

Various cytoskeletal molecules have been implicated in maintenance of the basal processes. The attachment of the basal endfoot to the pia is mediated by various receptors and extracellular matrix (ECM) components. Many of these molecules have been associated with MCDs, most commonly because the defective radial scaffold leads to impairments in the migration of neurons (Figure 2B). Knockout mice for Marcks (myristoylated alanine-rich substrate protein), an actin cross-linking protein, disrupted basal end feet and led to a disorganization of the radial scaffold, which resulted in displacement of progenitors and heterotopia (Blackshear et al., 1997; Weimer et al., 2009). A deletion in the regulatory region of *GPR56* [guanine nucleotide-binding protein (G protein)-coupled receptor 56], was found in patients with polymicrogyria surrounding bilaterally the Sylvian fissure including Broca’s area, the primary language area (Bae et al., 2014). Knockout mice show reduced cortical thickness and irregular cortical organization due to the defect in the progenitor proliferation (Bae et al., 2014). GPR56 localizes to the basal endfeet and binds to ECM components in the basal lamina, such as collagen III, which is considered to promote the proliferation of aRG and bRG (Singer et al., 2013). GPR56 functions together with α3β1 integrin, another major receptor of the ECM components (Jeong et al., 2013). The role of integrins in progenitor proliferation has been particularly highlighted in the context of BPs, and in particular bRG, in the gyrencephalic species (Fietz et al., 2010; Stenzel et al., 2014; Kalebic et al., 2019). Hence the disruption of the basal process is often linked to the perturbation of proliferation, in addition to migrational defects (Bizzotto and Francis, 2015; Kalebic and Huttner, 2020).

The most frequent cause of cobblestone lissencephaly are the mutations in genes which are required for the functional maturation of α-dystroglycan, another major ECM receptor (Devisme et al., 2012; Bizzotto and Francis, 2015). These genes include *POMT1* and *2* (Protein O-mannosyltransferase 1 and 2) (Beltran-Valero de Bernabe et al., 2002; van Reeuwijk et al., 2005), *POMGNT1* (Protein O-linked mannose *N*-acetylglucosaminyltransferase 1) (Yoshida et al., 2001), *LARGE* (LARGE xylosyl-and glucuronyltransferase 1) (van Reeuwijk et al., 2007), *TMEM5* (Transmembrane protein 5), *ISPD* (Isoprenoid synthase domain containing) (Vuillaumier-Barrot et al., 2012) and others. Finally, one-third of the cases of cobblestone lissencephaly are still unexplained, suggesting that other genes and pathways are involved (Devisme et al., 2012). Mutations in the transmembrane protein TMTC3 (transmembrane and tetratricopeptide repeat containing 3), which does not contain obvious functional connections to α-dystroglycan, have been found in patients with cobblestone lissencephaly (Jerber et al., 2016).

Mutations in the genes encoding for the components of the ECM can also lead to MCDs (Figure 2B). A notable example is *LAMB1* (laminin subunit β1) whose mutations were found in patients with cobblestone lissencephaly with severe cerebellar dysplasia, brainstem hypoplasia, and occipital encephalocele (Radmanesh et al., 2013). Enzymes involved in the production of ECM components have been associated with MCDs. ECE2 (endothelin-converting enzyme-2) has recently been found mutated in patients with periventricular heterotopia (Buchsbaum et al., 2020). Genetic manipulation in embryonic mouse neocortex and human cerebral organoids revealed that ECE2 is important for apicobasal cell polarity, apical belt integrity, actin and microtubule cytoskeleton dynamics and production of ECM components (Buchsbaum et al., 2020).

### Pro-proliferative Signaling

Various signaling pathways are involved in promoting proliferation of neural progenitor cells. Notch, Shh, Wnt, PDGF, FGF, ECM-integrin, ERK, Hippo, PI3K-AKT, and mTOR are some of the most notable examples (Penisson et al., 2019; Ferent et al., 2020; Kalebic and Huttner, 2020). Many of the components of those pathways have been implicated in MCDs. Among the receptor tyrosine kinases, fibroblast growth factor receptors (FGFRs), are particularly relevant associated with the syndromes characterized by brain malformations. Apert syndrome, in which brain malformation is considered secondary to the cranial abnormalities, is caused by mutations in FGFR2 (Aldridge et al., 2010). Instead, a mutation in FGFR3, which leads to a constitutive activity of the receptor, causes thanatophoric dysplasia (Hevner, 2005; Pannier et al., 2009). This disease is characterized by a combination of cortical abnormalities, which affects most severely the temporal lobe (Hevner, 2005). Mouse model of the thanatophoric dysplasia successfully recapitulated the megalencephaly phenotype, but not the other abnormalities (Lin et al., 2003). Instead the ferret model recapitulated all the other phenotypes found in human patients, such as polymicrogyria (Masuda et al., 2015), periventricular nodular heterotopia (Matsumoto et al., 2017a) and leptomeningeal glioneuronal heterotopia (Matsumoto et al., 2018). Similarly to FGF signaling, insulin growth factor (IGF) signaling through IGF1R (type 1 IGF receptor) stimulates progenitor proliferation and mutations in IGF1R were detected in patients with brain overgrowth (Faivre et al., 2002; Joseph D’Ercole and Ye, 2008).

Notch signaling pathway plays an essential role in neurogenesis through the process of lateral inhibition (Fortini, 2009; Pierfelice et al., 2011). In mammalian neocortex, Notch ligands are expressed by neurons and intermediate progenitors which signal back to aRG and bRG (Hansen et al., 2010; Nelson et al., 2013). The different combinations of Notch signaling molecules are involved in maintenance and likely diversification of the progenitor pool (Nelson et al., 2013). Further, Notch signaling can be amplified by reelin, a glycoprotein secreted by Cajal–Retzius cells. In addition to controlling neuronal migration and neocortical lamination, reelin promotes symmetric proliferative division of radial glia modulating thus the rate of neurogenesis (Lakoma et al., 2011; Hirota and Nakajima, 2017). Mutation in *RELN*, the gene encoding for reelin, have been shown to be associated with autosomal recessive lissencephaly as well as various neuropsychiatric disorders, such as schizophrenia (Hong et al., 2000; Fatemi, 2001; Ishii et al., 2016).

Recent studies have shown that diverse forms of brain overgrowth are often caused by mutations in the PI3K (phosphatidylinositol-3-kinase)–AKT signaling pathway (Hevner, 2015). Activating mutations of PI3K-AKT can cause hemimegalencephaly, dysplastic megalencephaly, heterotopia, polymicrogyria, pachygyria, and focal aggregates of small undifferentiated cells. Mildly activating variants, that are usually constitutional or germline, are associated with diffuse megalencephaly with intellectual disability and/or autism spectrum disorder, while moderately and strongly activating variants emerge as mosaic mutations and they are associated with mosaic megalencephaly, hemimegalencephaly and focal cortical dysplasia (Lee et al., 2012; Poduri et al., 2012; Riviere et al., 2012; Hevner, 2015; Jansen et al., 2015; Alcantara et al., 2017; Dobyns and Mirzaa, 2019). Mouse mutants for *Akt3* and *Pten* (phosphatase and tensin homolog) showed significant enlargement of the brain, but could not recapitulate the phenotypes pertinent to cortical folding (Groszer et al., 2001; Tokuda et al., 2011). Interestingly, when *PTEN* was deleted in human brain organoids, the subsequent activation of the PI3K-AKT pathway and increased proliferation of neural progenitors led to an increase in organoid size and the onset of organoid folding (Li et al., 2017). Mutations in the small GTPase gene RAB39b are associated with macrocephaly, autism spectrum disorder and intellectual disability (Woodbury-Smith et al., 2017). Deletion of RAB39b promotes PI3K–AKT signaling and leads to increased progenitor proliferation and macrocephaly in mouse model and increased organoid size in human *in vitro* model (Zhang et al., 2020).

PI3K-AKT signaling inhibits the activity of GSK3 (glycogen synthase kinase 3), a fundamental regulator of radial glia polarity. GSK3 inhibition in mouse aRG disrupts the radial organization of cell processes impairing thus the scaffold system that allows neuronal migration and affecting the progenitor proliferation (Yokota et al., 2010). Similarly, chronic inhibition of GSK3 in human cortical organoids increased the proliferation of neural progenitors (Lopez-Tobon et al., 2019). Some cases of megalencephaly and polymicrogyria are caused by mutations in *CCND2* (cyclin D2), a protein implicated in the cell cycle progression and a target of GSK3 (Kida et al., 2007; Mirzaa et al., 2014a). *In utero* electroporation of mutant *CCND2* into embryonic mouse cortex resulted in an increase in neural progenitor proliferation, likely explaining the brain enlargement of human patients (Mirzaa et al., 2014a).

Mammalian target of rapamycin (mTOR) signaling, also modulated by PI3K-AKT, is on the most important biological pathways and it is implicated in various diseases ranging from cancer to neurodevelopmental pathologies (Dobyns and Mirzaa, 2019). Mutations that affect mTOR are a common cause of focal cortical dysplasia and brain overgrowth along with the associated intellectual disabilities (Mirzaa et al., 2016; Marsan and Baulac, 2018; Kumari et al., 2020). Focal cortical dysplasia type II, which is the main cause of refractory epilepsy, is often caused by brain somatic mutations in mTOR kinase that lead to its hyperactivation (Lim J. S. et al., 2015). Indeed, overexpression of mutant mTOR by *in utero* electroporation in mice disrupts neuronal migration and causes spontaneous seizures, whereas the inhibition of mTOR can supress the seizures (Lim J. S. et al., 2015). Somatic mutations in genes involved in mTOR pathway, that occur in an early cell cycle of neocortical progenitors can lead to a more detrimental phenotype, that is hemimegalencephaly (Sarnat and Flores-Sarnat, 2014). The pathological phenotype can be a consequence of either a rare disruptive event causing hyperactivation of the pathway, or through the collective effects of many common alleles (Reijnders et al., 2017). Such activation of the pathway typically leads to over-proliferation of neural progenitors and subsequent increase in brain size (Dobyns and Mirzaa, 2019).

The pathway PI3K-AKT-mTOR is particularly interesting as mTOR pathway seem to be enriched in bRG (Nowakowski et al., 2017; Andrews et al., 2020) and PI3K-AKT is highly upregulated upon forced proliferation of mouse BPs (Kalebic et al., 2019). This suggests that PI3K-AKT-mTOR might play a specific role in promoting proliferation of BPs in species with an expanded cortex, such as human, and that the perturbations of its activity could have particularly relevant consequences for the proliferation of human bRG. Hence, a better understanding of the cellular processes impaired by PI3K-AKT-mTOR mutation would be fundamental for providing early diagnosis and appropriate therapy based on pathway inhibitors.

### Cell Metabolism

Neocortical progenitor metabolism is emerging as an important player for progenitor proliferation and cortical expansion and its dysregulation can lead to various neurodevelopmental disorders (Namba et al., 2021). Mitochondrial activity has a fundamental role in regulating the proliferation of progenitors and the deficiency of mitochondrial function can cause MCDs. For example, Amish lethal microcephaly is caused by a mutation of the *SLC25A19* (Solute carrier family 25 member 19), coding for a mitochondrial thiamine pyrophosphate carrier, which is a coenzyme for α-ketoglutarate dehydrogenase that operates in the TCA (tricarboxylic acid) cycle, (Kelley et al., 2002; Rosenberg et al., 2002). Interestingly, it appears that glutaminolysis, which provides α-ketoglutarate, is required for the proliferation of neural progenitors. MCPH1, which can be located also on the mitochondria, interacts with VDAC1, an ion channel on the outer membrane, and stimulates the mitochondrial activity *via* glutaminolysis, increasing the mitochondrial calcium concentration (Journiac et al., 2020). When *MCPH1* is mutated, the defects in mitochondrial structure and metabolism lead to a reduction in cell proliferation and survival and finally to microcephaly (Journiac et al., 2020). Furthermore, glutaminolysis has been identified as the principal mechanism that the human-specific ARHGAP11B utilizes to promote BP proliferation (Namba et al., 2020). Together, this suggests that the glutaminolysis might have an important role in both brain evolution and the onset of MCDs. Enzymes involved in asparagine and serine synthesis have been also implicated in microcephaly (Ruzzo et al., 2013; Acuna-Hidalgo et al., 2014). For example, mutations in *ASNS*, which encodes asparagine synthetase, have also been identified in patients characterized by congenital microcephaly, intellectual disability, progressive cerebral atrophy and intractable seizures (Ruzzo et al., 2013). ASNS mutant mice have structural brain abnormalities, including enlarged ventricles, reduced cortical thickness, deficits in learning and memory (Ruzzo et al., 2013).

In addition, defects in fatty acid metabolism and transport have been shown to cause microcephaly. A microcephalic patient has been identified carrying a deletion of *BBOX1* (butyrobetaine-gamma 2-oxoglutarate dioxygenase 1), that encodes an enzyme involved in carnitine synthesis (Rashidi-Nezhad et al., 2014). Carnitine shuttle system is required for the transport of long-chain fatty acids into mitochondria. Cpt1a (carnitine palmitoyltransferase 1A), an essential protein in the carnitine shuttle system, is involved in maintaining the proliferation of neural progenitors (Knobloch et al., 2017). MFSD2A (major facilitator superfamily domain–containing 2a) is a transporter required for the uptake of docosahexanoic acid (DHA) in the brain. Inactivating mutations in this gene cause a lethal microcephaly syndrome due to inadequate uptake of the essential omega-3 fatty acid (Guemez-Gamboa et al., 2015). Lipid metabolism might be particularly important for the proliferation of BPs since palmitoylation, a reversible lipidation with profound roles in the development and function of the nervous system (Fukata and Fukata, 2010), has been recently shown to be required for maintaining the proliferation of BPs in the human cortical tissue (Kalebic et al., 2019).

## Discussion

Genetic linkage studies have been the basis for understanding the mechanisms underlying the MCDs so far. In contrast, their diagnosis and classification are mainly based on the neurological outcomes at the tissue and organ levels. Hence, understanding the cell biological context in which these molecules operate is key in order to improve the mechanistic knowledge and better bridge between genes and phenotypes. This is particularly relevant for the neural progenitors, as their cell biology often appears to be the *fons et origo* of many MCDs. Disrupted proliferation of neural progenitors can lead to microcephaly and macrocephaly, whereas impaired polarity and/or detachment from the apical and basal pole of the tissue can lead to lissencephaly or heterotopia. The situation, however, is more complex since various cell types, principally migrating neurons, can have a dominant role in specific MCDs. It is further known that mutations in the same genes can exert different roles in progenitors and neurons. Further complexity of MCDs is characteristic of the genetic and phenotypic levels. MCDs can have both monogenic and polygenic causes, whereas genetic mutations can have both convergent and divergent relations with the final phenotypes (Klingler et al., 2021). In addition to genomic mutations, there are various environmental factors that have been implicated in MCDs: alcohol, stress, nicotine, cocaine, hypoxia, and various viral infections, notably ZIKA and SARS-Cov-2 (Luhmann, 2016; Sarieva and Mayer, 2021). To tackle the mechanisms through which all of these contribute to the onset of MCDs, it is important to understand the affected features at the cell biological level. For example, prenatal exposure to alcohol can cause impaired neuronal migration, reduced proliferation and altered morphology of neural progenitors (Hirai et al., 1999; Mooney et al., 2004; Cuzon et al., 2008; Luhmann, 2016). Particularly strong effects were observed on production and migration of GABAergic interneurons (Cuzon et al., 2008; Boa-Amponsem et al., 2020). ZIKA virus causes microcephaly by reducing neural progenitor proliferation and inducing cell death. The phenotypes were recapitulated in both mouse models and cerebral organoids with the organoids revealing a potentially key role of bRG in this process (Faizan et al., 2016; Qian et al., 2016; Watanabe et al., 2017; Sutarjono, 2019).

We have above listed key cell biological features in neural progenitors implicated in the emergence of MCDs and we can broadly split them into two groups: (1) fine regulation of mitosis and (2) maintenance of the correct cell polarity (Figure 2). Mitosis and notably the orientation of the mitotic spindle are important in determining the fate of the daughter cells, whereas the correct polarity (i) allows the access to the pro-proliferative signals at the apical and basal sides of the tissue and (ii) it enables the neuronal migration along the basal fibers of aRG and bRG. It is important to note that in addition to those principal groups various other cell biological processes are implicated. We have discussed some of the key signaling pathways and the emerging role of the cell metabolism in the onset of MCDs. Importantly, various molecules involved in the regulation of gene expression have been implicated in the emergence of MCD. Transcription factors, notably PAX6, TBR2, EMX2, FOXG1, ARX, DMRTA2, and others (Mallamaci et al., 2000; Kitamura et al., 2002; Ligon et al., 2003; Baala et al., 2007; Asami et al., 2011; Baek et al., 2015; Urquhart et al., 2016), as well as epigenetic mechanisms, including histone modifications, chromatin remodeling and RNA-level regulation, have been shown to play an important role in the onset of MCDs (Qiao et al., 2016; Silver, 2016; Albert and Huttner, 2018; Doan et al., 2018; Gabriele et al., 2018; Franz et al., 2019; Ciptasari and van Bokhoven, 2020; Vaid and Huttner, 2020; Reichard and Zimmer-Bensch, 2021; Wilson et al., 2021).

In light of the fact that the BPs, and in particular bRG, are the key cell type implicated in the development and evolution of the human brain (Lui et al., 2011; Borrell and Reillo, 2012; Florio and Huttner, 2014; Dehay et al., 2015; Llinares-Benadero and Borrell, 2019; Kalebic and Huttner, 2020), it is becoming increasingly important to examine the emergence of MCDs particularly in the context of those cells. As discussed above, the recent efforts suggest that both in the context of mitosis and especially of cell polarity, BPs might have specific roles with a substantial influence on the emergence of MCDs. To better understand those mechanisms, it is vital to use model systems that faithfully recapitulate both the phenotypes of diseases and the cell biological underpinnings. Mouse, and to a much lesser extent rat, have been very helpful in understanding the basic molecular roles of genes involved in MCDs. They, however, do not faithfully recapitulate certain phenotypic characteristics of MCDs that are pertinent to an expanded cortex, such as cortical folding, nor some of the developmental features that might have a key role in the disease, such as presence of the outer subventricular zone with a high abundance of proliferative bRG. To overcome those limitations two main directions have been undertaken: (i) use of human *in vitro* models, such as cerebral organoids and (ii) use of animals with the expanded cortex, such as macaques and ferrets.

Cerebral organoids, together with the recently developed assembloids, are three-dimensional *in vitro* structures that show great potential for investigating complex human genetic states and modeling aspects of human neurodevelopmental pathologies (Quadrato and Arlotta, 2017; Chiaradia and Lancaster, 2020; Sidhaye and Knoblich, 2021). Organoid models of human MCDs can be generated either by introducing the disease-causing mutations into otherwise wild type IPSCs or by directly using patient-derived IPSCs (Adegbola et al., 2017; Iefremova et al., 2017; Li et al., 2017; Fiddes et al., 2018; Klaus et al., 2019; Lopez-Tobon et al., 2019; Dhaliwal et al., 2021; Kyrousi et al., 2021; Wegscheid et al., 2021), with the latter being instrumental in personalized medicine. Among the animal models, macaques are particularly interesting as they recapitulate many of the cell biological features of human BPs (Betizeau et al., 2013). First transgenic macaques, with mutated *MCPH1* and modeling human microcephaly, have been recently generated (Ke et al., 2016; Shi et al., 2019). Ferrets are ethically and logistically a more suitable model for various human diseases including some MCDs (Gilardi and Kalebic, 2021). Ferrets well recapitulate the key features of the expanded cortex such as folding and the presence of the outer subventricular zone and proliferative bRG (Borrell and Reillo, 2012; Kawasaki, 2014; Gilardi and Kalebic, 2021). Ferret models of MCDs can be generated through a pharmacological or genetic manipulation, with the latter being possible through *in utero* electroporation and transgenesis. Pharmacological inhibition of mitosis is used to generate a ferret model of cortical dysplasia (Noctor et al., 1999, 2001). *In utero* electroporation of ferrets (Kawasaki et al., 2012, 2013; Kalebic et al., 2020), which has been used to deliver human-specific genes and thereby further enforce ferret’s potential to model human brain characteristics (Kalebic et al., 2018), has been used to model lissencephaly (Shinmyo et al., 2017) and thanatophoric dysplasia (Masuda et al., 2015; Matsumoto et al., 2017a,b, 2018). Transgenic ferrets with a germline knockout of *Aspm* were generated as a model for microcephaly (Johnson et al., 2018). In addition to recapitulating the human microcephaly better than the mouse modes, *Aspm* KO ferrets show a displacement of aRG to the oSVZ where they resemble bRG, suggesting an evolutionary mechanism by which ASPM regulates cortical expansion by controlling the ratio between aRG and bRG (Johnson et al., 2018).

Because bRG are emerging as important in both the aetiology of MCDs and the evolution of the mammalian brain, it is tempting to discuss the co-evolution of MCD genes and the mammalian brain evolution. This is particularly relevant for the genes associated with microcephaly that display a strong signature of adaptive evolution in primates, cetaceans, and other mammalian orders with expanded brains (Montgomery and Mundy, 2014; Doan et al., 2018). For example, *MCPH1* has been positively selected in the primate lineage (Pulvers et al., 2015), whereas in anthropoid primates (monkeys and apes), the rates of evolution of *ASPM* and *CDK5RAP2* are associated with variation in brain size (Montgomery and Mundy, 2014). However, a recent study could not detect any human-specific adaptive evolution of microcephaly genes (Pervaiz et al., 2021).

In conclusion, the multi-level complexity of MCDs is a key factor preventing a better link between the genes and phenotypes, which in turn is fundamental to provide the patients with better diagnostic and therapeutic perspectives. Addressing some of this complexity at the cell biological level of neural progenitor cells is highly needed. The current efforts in utilizing more suitable model systems, that faithfully recapitulate key cell biological features of the disease onset, are an important step forward in creating improved diagnostic and therapeutic options and the personalized approaches.

## Author Contributions

Both authors wrote the manuscript, approved it for publication, and agreed to be accountable for the content of the work.

## Conflict of Interest

The authors declare that the research was conducted in the absence of any commercial or financial relationships that could be construed as a potential conflict of interest.

## Publisher’s Note

All claims expressed in this article are solely those of the authors and do not necessarily represent those of their affiliated organizations, or those of the publisher, the editors and the reviewers. Any product that may be evaluated in this article, or claim that may be made by its manufacturer, is not guaranteed or endorsed by the publisher.
